# The benefits of and barriers to using a social robot PARO in care settings: a scoping review

**DOI:** 10.1186/s12877-019-1244-6

**Published:** 2019-08-23

**Authors:** Lillian Hung, Cindy Liu, Evan Woldum, Andy Au-Yeung, Annette Berndt, Christine Wallsworth, Neil Horne, Mario Gregorio, Jim Mann, Habib Chaudhury

**Affiliations:** 1 Gerontology Research Centre, Simon Fraser University, Room 2818, 2800-515 West Hastings Street, Vancouver, BC V6B 5K3 Canada; 2 University of British Columbia, Vancouver, Canada; 3 Vancouver Coastal Health, Vancouver, Canada; 4 Community Engagement Advocacy Network at Vancouver Coastal Health, Vancouver, Canada

**Keywords:** Dementia care, Robotics, Older adults, Scoping review

## Abstract

**Background:**

Given the complexity of providing dementia care in hospitals, integrating technology into practice is a high challenge and an important opportunity. Although there are a growing demand and interest in using social robots in a variety of care settings to support dementia care, little is known about the impacts of the robotics and their application in care settings, i.e., what worked, in which situations, and how.

**Methods:**

Scientific databases and Google Scholar were searched to identify publications published since 2000. The inclusion criteria consisted of older people with dementia, care setting, and social robot PARO.

**Results:**

A total of 29 papers were included in the review. Content analysis identified 3 key benefits of and 3 barriers to the use of PARO. Main benefits include: reducing negative emotion and behavioral symptoms, improving social engagement, and promoting positive mood and quality of care experience. Key barriers are: cost and workload, infection concerns, and stigma and ethical issues. This review reveals 3 research gaps: (a) the users’ needs and experiences remain unexplored, (b) few studies investigate the process of how to use the robot effectively to meet clinical needs, and (c) theory should be used to guide implementation.

**Conclusions:**

Most interventions conducted have been primarily researcher-focused. Future research should pay more attention to the clinical needs of the patient population and develop strategies to overcome barriers to the adoption of PARO in order to maximize patient benefits.

**Electronic supplementary material:**

The online version of this article (10.1186/s12877-019-1244-6) contains supplementary material, which is available to authorized users.

## Background

To-date, healthcare settings in Canada and worldwide are under tremendous strains from the rapidly growing demand associated with the aging population and chronic conditions, such as dementia. The public expects healthcare organizations to keep pace with the changing societal needs and serve the elderly population with compassion and good care. Clinicians and care workers in hospitals and care facilities face challenges in providing good care for the growing numbers of people with dementia who may also have complex medical and mental health needs. In the hospital setting, research has shown that behavioral and psychiatric symptoms are common in people with dementia, affecting 75% of those with dementia at some point during their stay in acute care, which often leads to their being prescribed antipsychotic drugs [1]. Given the complexity of providing dementia care, adopting and integrating technology into practice could be seen as an important opportunity; however, it can also be perceived as a significant challenge.

Researchers and scientists have been exploring ways to utilize robotic technology to aid in the care of older adults. A few robots (e.g., Physically-Assistive Robots, PARs) were made to perform physical tasks, such as body lifting. Others such as social robots (or called Socially-Assistive Robots, SARs) were created to support the social and psychological needs of the elderly. Social robots may serve multiple functions such as affective therapy, cognitive training, social facilitator, companionship and physiological therapy [2]. Specifically, the social robot - PARO (a baby harp seal robot) was designed as a pet therapy for older people with dementia [3]. We are interested in PARO because it has been commercialized and used in care settings for more than a decade in multiple countries. Also, there has been more research conducted on PARO compared to other animal-like robots [2].

Real life animals offer benefits in supporting the well-being of the older people with dementia, but animals are not always amenable to care settings [4, 5]. Some people may be allergic to pet dander, or be afraid of animal bites. Robotic pets require less care and are safe to use. PARO has demonstrated benefits in reducing stress, anxiety, and antipsychotics use among older people with dementia [6–8]. Although there is a growing evidence base indicating the benefits, resistance and antipathy to using the social robot in care settings are persistent [9]. There is a need for gaining an in-depth understanding towards the application of PARO, i.e., what worked, in which situation, and how. While advancements in artificial intelligence offers new possibilities to support and improve dementia care, the uptake of robotic technology has remained low in hospital and other care settings [10]. At present, there has been no comprehensive review performed to examine the effectiveness of the social robot PARO and how PARO can be used to its full potential and to help meet the pressing challenges clinicians face in everyday clinical practice.

## Methods

This review aims to map out the empirical evidence on the key benefits of PARO, and to identify barriers that may impede the adoption of this social robot. The questions guiding this review are: What has been reported in the literature regarding the benefits of PARO in dementia care? What are the barriers to adopting PARO in the care setting? A scoping review is appropriate because it provides an overview of relevant literature in a field that is under-developed and to identify the key themes and contexts within a research topic [11].

Following the steps outlined by Joanna Briggs Institute, this scoping review involved five stages: (1) conducting broad searches, (2) refining selection criteria, (3) reviewing search results, (4) mapping literature, and (5) summarizing results [12]. Our project team consists of: patients (*n* = 2) and families (*n* = 3), two physicians, an occupational therapist and a nurse researcher. The search strategy involved identifying published journal articles and grey literature to cover the breadth of the available literature that reported the benefits of and barriers to using the social robot PARO in care settings. The search began in June 2018, and the latest search was conducted in September 2018. We included relevant literature regardless of methodological quality because majority of the studies in the existing literature have small sample size and/or exploratory. The review and analysis procedures were as follows:

1. Conducting broad searches to identify potentially relevant literature: The first four authors independently conducted the literature searches and screened titles, abstracts, and references. We undertook a wide range of literature searches using the following databases: MEDLINE, AgeLine, PsycINFO, and Cumulative Index to Nursing and Allied Health Literature (CINAHL). A university librarian was consulted. We looked at literature written in English from year the 2000 through September 2018. Search terms included: social robot, PARO, Alzheimer disease and dementia. Also, we searched Google Scholar and checked the references cited in relevant publications.
2. Refining selection criteria: Inclusion and exclusion criteria were applied to select articles. Duplication was removed. Articles were included if they: (i) focused on older people with dementia, (ii) targeted effects of PARO, and (iii) were studied in care settings (e.g., nursing homes, hospitals, and day care). Both quantitative and qualitative studies were included. Records were excluded due to: absence of any focus on older people with dementia, did not report PARO, was conducted outside a care setting (e.g., at home). A bibliographic reference management tool, Mendeley was used to ensure that all references and articles were systematically accounted.
3. Reviewing search results: Three authors (blinded for review) read the included articles to gain a preliminary sense of concepts of the whole. Afterwards, we developed an initial coding framework to code deductively while remained open to concepts that emerged inductively for new codes. A data analysis software, NVivo12 was used to conduct coding for full-text review in selected articles. The first three authors conducted content analysis [13].
4. Mapping literature according to conceptual areas of interest: We mapped the papers by domains: author and country, setting, participants, research design, measures, benefits, as well as barriers. See Additional file 1: Summary of included studies. In research meetings, patient and family partners in the research team took part in analyzing the extracted data sorted according to potential themes. We compared and discussed interpretations to resolve conflicts. The coded data were then evaluated, refined and collated into categories to develop the final themes.
5. Summarizing results: Three authors (blinded for review) wrote the first draft of the manuscript to summarize the results. All authors critically reviewed and participated in manuscript revisions.

## Results

**Fig. 1:**
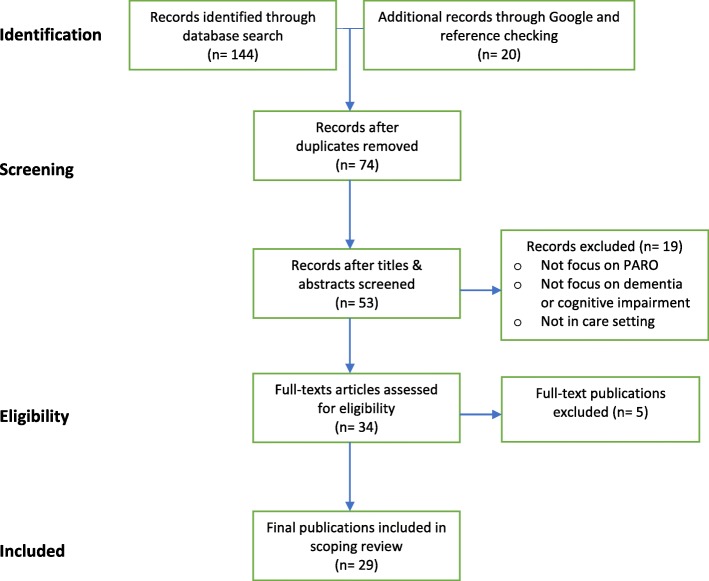
Flow Diagram for the scoping review process

The database search yielded 144 publications and an additional 20 from reference checking and Google Scholar search. After screening and inclusion assessment, thirty-four papers were assessed for full-text review. Five more articles were excluded due to content not relevant to the review questions. A total of 29 publications (*n* = 29) were included in the final review. Figure 1 shows the review flow diagram.

**Fig. 2:**
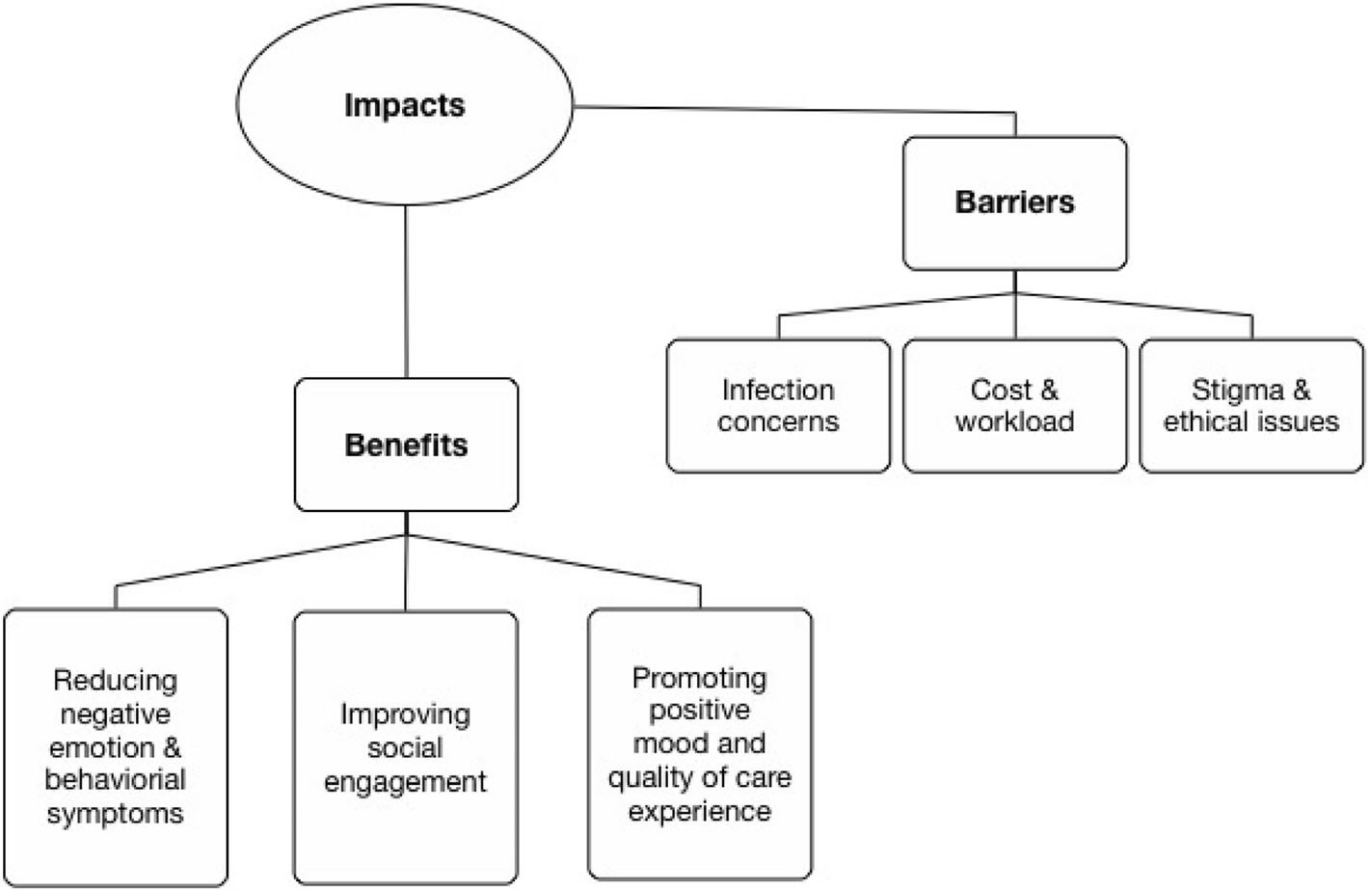
Final themes

Of the included publications (*n* = 29), 24 items with quantitative experiential designs reported positive outcomes. Common outcome measures were agitation, anxiety, depression, loneliness, cognition, and quality of life. The majority of studies’ scope was relatively small and exploratory. A recent Australian study with 415 older people with dementia from 28 long-term care facilities was an exception [7]. Most research reported the use of PARO in nursing homes (*n* = 25). More publications were authored in Australia, US, and Japan. Only one Canadian study (*n* = 3 participants) was found [14]. Only one study reported family perspective [15] and staff experience [11]. Content analysis [13] identified reported benefits of and barriers to the use of PARO. Our analysis serves to identity the key benefits (some of them overlaps and interacts) and core barriers. See Fig. 2 for the final themes.

### Benefits

Key benefits include: reducing negative emotion and behavioral symptoms, improving social engagement, and promoting positive mood and quality of care experience. Table 1 shows the benefits of PARO reported in publications.

**Table 1:** Benefits of PARO reported in included papers

Authors, setting & country	Reducing negative emotion and behavioral symptoms	Improving social engagement	Promoting positive mood and quality of care experience
Bemelmans et al., [16] Long-term care, Netherlands	+	+	+
Bemelmans et al., [17] Long-term care, Netherlands	+	+	+
Iacono & Marti, [18] Long-term care, Italy		+	
Jones et al., [19] Long-term care, Australia		+	+
Jøranson et al., [20] Long-term care, Norway	+	+	+
Jøranson et al., [21] Long-term care, Norway		+	+
Jøranson et al., 2015 [22] Long-term care, Norway	+		+
Kidd, Taggart, & Turkle, [23] Long-term care, US		+	
Lane et al., [24] Long-term care, US	+		+
Marti et al., [25] Long-term care, Italy	+	+	+
Moyle et al., [26] Long-term care, Australia	+		+
Moyle et al., [27] Long-term care, Australia	+		
Moyle et al., 2017, 2018 [7] Long-term care, Australia	+		+
Moyle et al., [15] Long-term care, Australia	+		+
Moyle et al., [11] Long-term care, Australia	+		+
Petersen et al., [6] Long-term care, US	+		+
Robinson et al., [28] Long-term care, New Zealand	+		+
Robinson et al., [29] Long-term care, New Zealand			+
Roger et al., [14] Long-term care, Canada	+	+	+
Šabanovic et al., [30] Long-term care, US		+	+
Sung et la., [31] Long-term care, Taiwan		+	+
Takayanagi et al., [32] Long-term care, Japan		+	+
Thodberg et al., [28] Long-term care, Denmark			+
Valentí Soler et al., [33] Long-term care, Spain	+		+
Wada et al., [34] Long-term care, Japan		+	+
Wada et al., [35] Day care, Japan		+	+
Wada et al., [36] Long-term care, Japan		+	+

### Reducing negative emotion and behavioral symptoms

One of the common targets for interventions in dementia is alleviating negative emotions and reducing behavioral symptoms. A recent Australian RCT compared PARO with a plush toy found a statistical significant but modest effect in reducing behavioral and psychological symptoms of dementia [7]. Other studies in Norway, US, and New Zealand also found that the social robot helped in the reduction of physical and verbal agitation [16–20]. PARO was also found to improve anxiety and improve depressive symptoms [6, 21, 22]. Evidence also indicated that the utilization of PARO reduced the use of psychotropic medication [19, 22–24], and combined with reduced wandering may reduce the falls risk [11]. These improvements suggested that the robot may result in reducing staff stress and caregiver burnout [25]. Some studies have reported the benefits in psychological and behavioral symptoms of dementia were more pronounced in those with less cognitive impairment [26, 27], and in individual as opposed to group settings [11]. Other studies showed older people with moderate and with severe dementia had a significant effect with PARO [21, 24]. The evidence was inconsistent and indicated the need for further research. It is also important to point out that the reporting of stages of dementia can be problematic in the literature as different cutting scores and scales were used in different studies.

Despite these positive findings, it was noted that staff in the residential care setting were challenged to use PARO effectively to provide care due to restricted work routines [28]. The experimential design of research prescribed fixed intervention time and dose, which did not always match clinical needs of residents in the care setting. In a staff experience study, PARO was reported to have many benefits and staff found it useful and practical for people with dementia to use [11]. Not every older person wanted to interact with PARO. One research reported that 1 out of 10 persons refused to interact with PARO [21]. Other studies did not report refusal rate.

### Improving social engagement

PARO has been found to improve social engagement in individuals with dementia, increased activity participation, and promote more spontaneous communication [22, 25, 27, 29]. PARO helped to improve both verbal and visual engagement [7] in social interactions. In a study, PARO was utilized to facilitate conversations between the individual with a therapist [30]. In another study, PARO was highlighted to work as an ice-breaker between staff and residents, a social mediator or an impetus toward social interactions between residents [19, 31]. A U.S. study reported PARO’s positive effects on the activity levels of older people with dementia grew over 7 weeks, suggesting Paro offered more than “novelty effect” [29]. A study in Taiwan showed short-term interactions significantly improved the communication and interaction skills of participants in residential care [32]. When PARO was compared with a stuffed animal in Japan, participants talked more frequently to PARO and showed more positive emotional expressions with PARO [21]. Rather than reducing human contact, the researchers found introducing PARO may increase willingness of the staff members to communicate and work with elderly people with dementia, especially those with moderate dementia [21].

A staff experience study in Australia commented that PARO provided a sense of belonging and warmness: “when I saw them interacting with it… you saw their loving personality came back” [11]. Also, staff perceived that PARO gave older people with dementia (including males) confidence to talk with others around them: “The men don’t really tend to take with the babies a lot, whereas they did with the seal” [11]. In a storytelling study, participants in the PARO group not only spoke more words, but also were more articulate on the creation of story characters, setting and story [37]. It was suggested that improved communication contributed to more calmed behaviors and improved mood [19, 25], and reduced loneliness [14]. Some studies indicated improved social engagement persisted for longer than a year [3, 33].

### Promoting positive mood and quality of care experience

Multiple studies have found improvements to positive emotions and behaviors in individuals with dementia interacting with PARO. PARO has been noted to help individuals become more active, smiling, relaxed and comfortable, more likely to laugh, and to have brighter facial expressions [23, 32, 34]. It has also been found to improve participants’ mood and the quality of care reported by caregivers, as well as the level of comfort observed by families [7, 35]. Increased quality of life and pleasure scores with the use of PARO have suggested improvement in care experience [7, 26]. Other studies have demonstrated positive effective in sleep [16, 19] and pain medication use [6]. In a quality of life research, the participants who spent time with PARO (intervention group) showed to have a sustained improvement in quality of life, in comparison to a worsening trajectory in the control group [23, 24]. The intervention group used significantly less psychotropic medication compared with the control group. Family interviews in a study [15] found families reported PARO was something to love, offered meaningful stimulation, and companionship. Family comments included: “Everybody I saw with it, it certainly seemed to lighten their mood” and “I think for her it’ a companion, somebody to talk to, she’s not lonely”. Overall, evidence showed PARO may help to stimulate memories, promote positive mood and quality care experience [19].

### Barriers

While the social robot PARO offers technological opportunity in supporting dementia care and managing difficult behavioral symptoms, the adoption of PARO in care setting remains low. Key barriers to the adoption of the technology include: cost and workload, infection concerns, and stigma and ethical issues.

### Cost and workload

One identified barrier to the uptake of social robot is cost and added workload to staff. Since PARO was often used individually or in small groups, the initial cost of purchasing a unit was brought up as a barrier to use in care settings [15, 17, 20, 32]. The current cost of the robot is US$6000. Although there is government support in some countries such as U.S. and Japan (as PARO is certified as a therapeutic medical device), most healthcare organizations in other countries have to purchase their own. The high cost can lead to a concern in innovation dissemination, fair distribution, and equity in the robotic use [10]. Currently, universal access by fair opportunity to assistive technologies is an ideal but not a reality. A few studies also highlighted ongoing maintenance, cleaning and repair can be an added cost [17, 36]. Additionally, staff education and skill at facilitation and application have been identified as important aspects of using the robot so PARO may be perceived as additional workload for caregivers and staff [15, 32, 35]. A few studies also brought up a concern that with patients in distress and frustration, PARO may be damaged and may not be able to sustain in shared use with multiple residents within care facilities [3, 22].

### Infection concerns

Another key consideration is infection prevention and control. Studies highlighted that it can be difficult to keep PARO’s fur clean [11, 38], and that the fur covering is not designed to be regularly removed or machine washed, which may post a concern especially to individuals who are immunocompromised [3, 22]. To keep to a minimize the spread of pathogens, the recommended protocol involves cleaning PARO between contact with different users [35], which may be seen as an added workload to staff in facilities. In a UK study at a general hospital over 9-month of time, PARO was used with a hospital infection control protocol and found to be within the benchmark threshold for cleanliness [9]. The authors commented, “However, during this study the time allowed for cleaning in the cleaning protocol was considered by the staff to be long and onerous. This had the potential to limit the use of PARO by affecting perceived workload” (p. 39).

### Stigma and ethical issues

The stigma of interacting with a robot animal was another concern identified by caregivers and staff. Some authors raised the ethical question that the use of robots in dementia care creates risk of infantilizing and dehumanizing care [10]. Research noted that individuals might feel as if they are being treated like children, and the robot being seen as “toylike” [11, 15, 35]. Some cases described individuals as appearing embarrassed about interacting with PARO especially in front of others, and this might have influenced their reactions [39, 40]. It was noted that this might be of particular concern to men, who seem to respond less positively to PARO in some studies [40, 41]. As previously mentioned, however, male residents in another study responded positively with PARO [11]. This suggests the gender factor should be further investigated.

In some cases, interventions with PARO caused negative emotional responses, including anger, wandering, fearfulness, and agitation [17, 20, 35]. Studies postulated that some individuals may have had past negative experiences with animals, therefore, consideration should include the person’s biography, particularly their like and dislike of animals [42]. It was noted that when using PARO, staff should uphold a person-centred approach, as just because the resident liked PARO 1 day does not mean that he or she will enjoy it the next [26]. Some staff and family raised concern that PARO’s vocal sounds and movements could be distressing [38]. Trying to engage patients who were not interested could lead to increased agitation [36, 40]. PARO was found to not have the option to easily turn off because its hidden switch between the split tail fins; older people with dementia did not know how to turn the robot on or off [43]. Removing PARO was sometimes noted to be difficult [36]; after several weeks of removal of PARO, one study found increased depressive symptoms at follow up [27].

Some studies described situations where PARO appeared ineffective for some individuals or lost effect over time [34, 41]. The differences in how specific subgroups may respond to the robot remain unclear and need further research. A few studies noted engagement was less likely with males, and those who were more cognitively impaired tended to interact with PARO and not with other humans [6, 41]. For example, we do not know whether or not an individual’s previous positive experiences with animals could affect level of engagement [38]. The perceptions of PARO as a pet versus as a therapeutic tool might differ depending on cultural acceptance [42]. Regulating the robot as a medical device has disadvantages (e.g., keeping the price high and inequity of distribution) and advantages (e.g., safety regulations). Table 2 offers practical advice to draw on for stakeholders who are responsible for addressing barriers and ensuring safe, competent and ethical application.

**Table 2:** Key barriers and implications

Barriers	Implications
Cost and workload • High cost • Staff workload	Consider shared use of the robot to serve a larger group of population in care settings Involve healthcare professionals in co-developing strategies to fit workflow, improve effectiveness, and meet clinical needs
Infection concerns • Sharing and spreading disease	Engage infection control practitioners, leadership, and frontline to develop practice guidelines and protocols Provide training and ongoing support to ensure staff understand how to clean the robot and follow infection prevention procedures
Stigma and ethical issues • Robot replacing human • Reducing human contact • Objectification • Infantilizing • Deception	Avoid the ‘human vs robot’ thinking, technology should complement but not replace the care provided by clinicians Learn the person’s biography and apply a person-centered approach Work with frontline and leaders in organizations to clarify the role of the robot and find out how the robot can be used most effectively Investigate if the robot works with people with different stages and types of dementia, gender, ethnic and cultural backgrounds

## Discussion

In this scoping review, we identified key benefits of and barriers to the adoption of social robot PARO in care settings. Our findings suggest that while existing research studies demonstrated positive benefits of the social robot PARO in supporting the psychosocial needs and care experiences in dementia care, there is a need to produce more robust knowledge to support effective uptake. There is a need to explore the complexity of technology use in a sustained manner. For example, process evaluation and qualitative studies are required to gain a better understanding of what aspects of the psychosocial intervention work and do not work, for whom, and in what situations [36]. Our analysis identified three major research gaps: (a) the first-person perspective of patients’ experiences and clinical needs remain unexplored, (b) few studies investigate the process of how to use the robot effectively in different situations to meet clinical needs, and (c) there is a need to apply relevant theory or conceptual frameworks to have a grounded understanding of the robot-human interaction and guide effective and appropriate application.

### Users’ perspective

The low uptake of social robot for dementia care could be a result of gap in unmet users’ needs and structural limitations in healthcare organizations. The users may include clinicians, patients, families, and policy-makers and healthcare leaders. Our findings show previous research was more researcher-centered. There is a need to shift this research paradigm to be more patient–oriented and user-centered [10]. The first person’s perspective about what matters and their priority needs have not been explored. Innovative ways such as video methods [44, 45] that accommodate memory problems and enable active participation should be utilized to explore patients’ perspectives. Another important gap is the frontline clinicians’ perspective. Clinicians, families, policy makers, and organizational leaders need to be engaged to identify strategies to enable successful translation of robotic technology. Future research should pay more attention to patients’ experiences and clinicians’ practice to ensure technology use adds values to the clinical care. For example, in a recent study of older adults’ perspective, the users considered appearance, functionalities and social capabilities to be important elements of social robots [46]. As reported by Lourida et al. (2017), a recent review on implementation of evidence-based dementia care intervention, they found organizational factors, such as time, workload, managerial support, knowledge, attitude, staff engagement are important factors for successful implementation of evidence-based practice in dementia care. Without user engagement and meaningful collaborations, working in silos is unlikely to fully realize the potential benefits of any robotic devices to meet the current and future challenges that people in healthcare face.

### The process of how

Findings of this review indicate a paucity of research focusing specifically on the process of implementation of the robot in healthcare institutions. More research is needed to investigate the implementation process - how to engage knowledge users to achieve greatest impact. [47] The technology adoption lifecycle is a helpful model that describes the process of adoption over time involves groups of innovators, early adopters, early majority, late majority, and laggards. [48] PARO can be moving in the transition between early adopters and majority. Therefore, it is important to fully understand barriers to adoption, patients’ experiences and pressing clinical issues to support adoption for practice change. The adoption of PARO in Denmark is a good example. [19] Over 80% of the local care institution in Denmark are currently using PARO. PARO is recognized as a therapeutic tool for care professionals; the Danish Technological Institute (a knowledge mobilization organization) provides a training program on PARO use. Nursing staff in Danish facilities use PARO to promote residents’ sleep, improve mood, support social communications, reduce anxiety, aggression and agitated behaviors.

Most studies used statistical significance to identify effectiveness. We acknowledge that it is difficult to find a statistical significant analysis because it is too expensive to provide a large number of PARO robots. Using statistical significance and outcome-based approach to assess impact are inadequate as they do not take into account the multiple interactive factors that may influence the human-robot interaction. For example, shared values and purpose of the local team and organization may affect the attitude and behaviors of clinicians in using the robot for care. What is clinically significant (what matters to patients, families, and clinicians) may not be captured by statistical significance. We also found that training and education were not adequately used in clinicians and stakeholders to facilitate uptake. Organizational and structural factors that may influence technology adoption but were not investigated and reported. Future work should report implementation process and identify facilitators or strategies that were effective to overcome barriers to successful adoption.

Healthcare funding models and constraints on healthcare funding can play a substantial role in social robot adoption. For example, PARO in the US is a medical device and billable to Medicare. PARO can be prescribed as an alternative therapy in the US. Physicians, psychologists, and nurse practitioners have their reimbursement rates. [49] However, this is not the case in Canada even though Canadians have universal access to most healthcare services. Fair opportunities to access technology use should be an important goal for governments. For social justice and equity reasons, there is a need to develop funding structure to make technologies affordable to those who need them. It is necessary to understand what (e.g., resources and skill training) is needed to address issues to clear the way for staff to work effectively with robotic technology in clinical practice.

### Apply theory and embrace complexity

Almost all of the available literature did not apply theories to guide the intervention research. Future research will benefit from using theories/models to understand how the social robot may meet the psychosocial needs of people with dementia. Also, knowledge translation theories can be utilized to contextualize drivers, barriers as well as conditions conducive for effective application. Innovative methods should be used to shed light on the complex dynamics of implementation content in dementia care [36]. Organizational leaders, managers, educators, physicians, nurses, therapists, care staff, families, and patients may each have interests related to their role in the care settings. It is pivotal to consider context as a complex adaptive system; the interplay between interventions, implementation strategies and context are interacting components of a complex system [50].

### Strengths and limitations

This review offers a meaningful contribution as our findings have implications for stakeholders with responsibility for applying technology in supporting dementia care. We followed the established guideline by the Joanna Briggs Institute to ensure the entire review process is rigorous and transparent. Our team analysis included patient and family partners, as well as an interdisciplinary team to ensure quality. The screening and article selection was conducted independently by team members in multiple disciplines, including physicians, an occupational therapist, and a nurse researcher. The diverse perspectives in our project team enrich the analysis and add credibility to the review.

This review has several limitations. Literature published in other language was not searched. There is relevant literature on the social robot published in other languages but were not included in the review. Our search strategy may have been biased toward health and sciences. Searching other technological databases may have yield additional articles. We did not contact experts for checking additional articles we may have missed.

## Conclusions

This scoping review has mapped the reported benefits of using the social robot PARO in supporting older people with dementia within care settings and revealed a paucity of evidence to inform how the social robot could be most effectively adopted to meet clinical needs. In previous studies, interventions evaluated have been primarily researcher-focused. Future research should consider deeper user involvement, including patients and families, frontline clinicians, policy makers and organizational leaders to co-design translation strategies for integrating technology into care. Lastly, there is a need to apply theory to understand how the social robot may meet the psychosocial needs of people with dementia.

## Additional file

Additional file 1: Summary of included studies (DOCX 75 kb)

## Data Availability

The raw data set is made available as an additional supporting file of this manuscript. Please see Additional file 1: Summary of included studies.

## Abbreviations

PARs: Physically-Assistive Robots
SARs: Socially-Assistive Robots
