# Supplementary material for: The benefits of and barriers to using a social robot PARO in care settings: a scoping review
Source: BMC Geriatr. 2019 Aug 23;19:232. doi: 10.1186/s12877-019-1244-6 (PMC6708202; doi:10.1186/s12877-019-1244-6)
Supplement: Supplementary file 1 — Summary of included studies (DOCX 75 kb) [file 12877_2019_1244_MOESM1_ESM.docx]

|  | **Author &**  **country** | **Setting** | **Participants** | **Research design** | **Measures** | **Benefits** | **Barriers & Implications** |
| --- | --- | --- | --- | --- | --- | --- | --- |
|  | (Bemelmans et al., 2015)  Netherlands | Psychogeriatric care | 71 participants (14 men) with  dementia in two groups:  therapeutic intervention and care support intervention | Supervised one-on-one  interaction with Paro or no intervention. Paro either served as a therapeutic or  a care support tool in 2  separate phases of the  study. Crossover study. Five sessions (~15 min)/  month for 2 months;  each month of therapy  was interspersed with  a control month. In the  therapeutic arm only,  additional sessions were  given when patient was in distress. | IPPA, mood scale, behavior rating scale | Therapeutic-related  interventions show an  increase of IPPA scores by  two points (P<0.01).  Care support intervention  showed no effect. | Attention should be paid to hygiene if the robot is to be used by multiple residents, and that in terms of practical use, storage of the robot and charging of the battery needs to be well organized and structured. |
|  | (Bemelmans et al., 2016)  Netherlands | Psychogeriatric care | 23 dementia patients, 22 females and 1 male | Paro was used according to individualized interventions, aiming at predeﬁned speciﬁc care problems, during a 3-week period. Selected residents were offered Paro once or twice a week. Three intervention types were applied, one for therapeutic purposes, one for facilitating daily care activities and one to support social visits. | Qualitative questionnaires & data in a registration form | In the majority of cases, care staff and patients considered the Paro interventions to be of added value for the care provided. | The intended effects of the use of Paro for each participating individual was made both clear and measurable. It clariﬁed what care workers could do with Paro and what to expect from its use.  Clear descriptions of the intervention goals per patient need to be formulated; otherwise Paro is used in a way not relating the intervention goals. |
|  | (Dodds, Martyn, & Brown, 2018)  UK | Hospital | The number of patients and staff involved not mentioned in the paper | To address the infection control concerns raised, this study offers cleaning and testing protocols: using biocide wipes | The cleanliness of PARO was determined using an adenosine triphosphate (ATP) luminometer | Over a nine-month period of using PARO in everyday clinical practice, using the cleaning and monitoring protocols developed in collaboration with the unit, PARO was within the benchmark threshold and complied with local and national IPC requirements. | Drying of PARO fur takes eight to 15 minutes in the clinical environment. Staff were taught the cleaning process and a recording system that monitored the use and cleaning of PARO was implemented. Nominated staff took responsibility for routine cleaning. |
|  | (Iacono & Marti, 2016)  Italy | Seniors’ residence | 6 women with an average age of 79.67 | The stories collected during the experimental sessions (story-making with Paro and another social robot - Sugar) | Narratives’ length, terminology, emotional content and coherence | The results show that interacting with Paro, unlike Sugar, stimulates storytelling, encouraging interaction among participants and supporting the construction of more emotionally intense content. | Cultural and gender differences were not taken into account in the present study that involved only women. Additional researches would be necessary to appreciate the impact of cultural and gender differences. |
|  | (Jones et al., 2018)  Australia | Nursing homes | 138 residents with dementia | Data from the PARO intervention-arm of a cluster-randomized controlled trial was used, which involved individual, non-facilitated, 15-minute sessions with PARO 3 afternoons per week for 10 weeks. | CMAI, RUDAS | Participants with severe agitation had poor response to PARO. Lower levels of agitation and higher cognitive functioning were associated with better responses. | Considerations should include the person’s biography, particularly their like or dislike of animals  When using PARO, staff should uphold a person-centred approach, as just because the resident liked PARO one day does not mean that he or she will enjoy it the next. |
|  | (Joranson et al., 2016)  Norway | Nursing homes | A convenience sample of 60 participants was recruited (67% women, age range 62-95 years) resulting in 30 in intervention group and 3o in control | Change in quality of life was assessed by local nurses through the Quality of Life in Late-Stage Dementia scale at baseline, after end of intervention and at 3 months follow-up. The scale and regular psychotropic medication were analyzed stratiﬁed by dementia severity. | BARS, CDR, QUALID, NPS | A significant effect was found among participants with severe dementia from baseline to follow-up showing stable quality of life in the intervention group compared with a decrease in the control group  The intervention group used signiﬁcantly less psychotropic medication compared with the control group after end of intervention. | Paro had a statistically signiﬁcant effect on QoL, but only for participants with severe dementia.  Researchers should further investigate effects of activities involving emotional robots on QoL also with respect to dementia severity NPS |
|  | (Jøranson et al., 2016)  Norway | Nursing homes | 30 participants (seven men) aged  from 62 to 92 years. All participants  had a dementia diagnosis | Supervised group  interaction with Paro.  Observational study. Two sessions (30 min)/  week for 12 weeks | Observed behavior as  seen in video recording | Participants with mild to  moderate dementia paid  more attention to Paro than  those with severe dementia.  Over the study period,  there was an increase in  interactions with other  participants and a decrease in  interactions with Paro. | Be aware of the challenges toward maintaining attention for people with severe dementia and consider shorter duration, fewer participants in the group setting, or, if necessary, individual Paro activity.  Future research should investigate topics of conversations in interactions. |
|  | (Jøranson, Pedersen, Rokstad, & Ihlebæk, 2015)  Norway | Nursing homes | 53 participants (20 men) aged 62–95,  with a cognitive impairment  (MMSE <25) or diagnosed  dementia | Supervised group  interaction with Paro  or TAU. Randomized  controlled trial. Two sessions (~30 min)/  week for 12 weeks | Cognitive status,  medication, BARS,  Norwegian version of  CSDD called CDR,  QUALID assessed  before (T0), after (T1)  and at 3-month follow-up  (T2) | Reduction in agitation in  Paro versus TAU from T0 to  T2 (P<0.05).  Reduction in depression  in Paro versus TAU from T0  to T2 (P<0.05).  In those with severe  dementia, quality of life  scores did not decrease in  Paro group from T0 to T2,  whereas they did in control.  No such difference was  found in mild to moderate  dementia group. | Enhancing quality of life in persons with severe dementia should be highlighted more in practice in terms of the importance for nurses and other staff to create engaging activities for this vulnerable patient group. |
|  | (Kidd, Taggart, & Turkle, 2006)  US | Nursing homes | 23 residents | Visited each home once every two weeks over 4 months for a total of 8 visits to home A and 4 visits to home B. During each session, we interacted with multiple groups of residents. One of three things happened: the Paro was brought out, placed on the table, and turned on; the Paro was brought out, placed on the table, and remained turned off; or the Paro was not brought out. | Social Interaction Questionnaire | Paro did effectively evoke memories of pets. In fact, many conversations about Paro turned into discussions about other animals, often cats & dogs.  Increase in social interactions. This effect is increased in the presence of caregivers or experimenters who are willing to participate in the interactions. | Paro presents itself as a baby seal. Residents have expressed their desire to put Paro in water and see if it can swim.  Paro does not easily turn on and off because its switch is  hidden between its split tail ﬁns and residents could not master it.  Some residents simply felt that “toys are for kids” and that  playing with them is inappropriate. |
|  | (Lane et al., 2016)  US | Veteran residential  care | 23 participants (all men) aged 58–97  years, 19 had been diagnosed  with dementia | Supervised one-on-one  interaction with Paro. Pilot  study. Three sessions (>5 min)  across 1 year | Behavior (assessment  form designed by authors of study—  no formal name)  Assessments made  before, during and after  interaction | 1. Increase in observed  positive affective and  behavioral indicators (e.g.,  bright affect, interacting with  others, calm).  2. Decrease in observed  negative affective and  behavioral indicators (e.g.,  anxious, sad and yelling).  3. Those who best  responded to Paro were  calm and approachable at  the before interaction | Paro is best presented to residents who are relatively calm and approachable  Not particularly useful when offered to a veteran in acute distress or in the midst of a catastrophic reaction  Overall magnitude of change noted by study staff appeared greater when looking at the pre–post difference for positive mood and behavioral indicators versus negative ones |
|  | (Marti, Bacigalupo, Giusti, Mennecozzi, & Shibata, 2006)  Italy | Nursing home | One patient | Case study: Paro is given to patients both in pre-planned therapeutic activity but also in critical situations, in order to contain unexpected behavioural disturbance episodes. Patients were filmed in everyday life situations with and without Paro. | Ethnographic observation of the activity in two different contexts: dyadic relations between the patient and the robot, and triadic relations involving also the therapist | In addition to stimulating positive emotion, Paro worked as a social mediator and allows the therapist to negotiate with the patient a common emotional ground | The relationship between the patient and the robot can constitute a “leverage point” for the therapist to open a communication channel with the patient.  Research on Social Robot should focus on the design for the dynamics that generate from the aesthetical, perceptive and emotional experience of interacting with the robot |
|  | (Mervin et al., 2018)  Australia | Long-term care | 415 residents, all aged 60 years or older, with dementia | Three groups: Paro, plush toy, usual care. Investigated the incremental cost per Cohen-Mansﬁeld Agitation Inventory Short Form (CMAI-SF) point averted from a provider’s perspective. | The incremental cost per Cohen-Mansfield Agitation Inventory Short Form (CMAI-SF) point | Both a plush toy and the PARO are cost-effective psychosocial treatment options for agitation. Cost of Paro is lower than values estimated for psychosocial group activities and sensory interventions | Not just the cost of the robot but also the cost of staff training for effective use |
|  | (Moyle et al., 2013)  Australia | Nursing homes | 18 participants, aged >65 years, with dementia | Supervised group  interaction with Paro or  reading group. Randomized  controlled trial. Three sessions (~45 min)/  week for 5 weeks | Modified QOLAD, RAID,  AES, GDS, Revised  Algase Wandering  Scale–Nursing Home  version and OERS | 1. The Paro group had  higher QoLAD and OERS Pleasure  scores following  the intervention.  2. The Paro group had  reduced OERS-Anxiety  and OERS-Sadness scores  following intervention. | Paro was left to the people with dementia, with no facilitation. To ensure Paro’s potential, developing the effective ways to use it is the next step. |
|  | (Moyle, Jones, et al., 2017)  Australia | Long-term care | 415 participants (101 men) mean  age 85 years. All participants were  diagnosed with dementia | Free one-on-one interaction  with Paro switched on,  Paro switched off or  TAU. Cluster-randomized  controlled trial. Three sessions (15 min)/  week for 10 weeks | Video observations (at  baseline and weeks 1, 5  10 and 15) and CMAI (at  baseline and weeks 10  and 15) | 1. Participants in Paro switched on group were more verbally and visually engaged compared with Paro switched off group.  2. Both Paro switched on  and switched off groups  had reduced neutral affect  compared with TAU group.  3. Paro switched on was  more effective than TAU  at improving pleasure and  agitation. | Helps patients during sun downing, staff felt that residents were often distressed but unable to express what was concerning them, and Paro offered opportunities to distract residents during these times. |
|  | (Moyle, Bramble, Jones, Murfield, & Bowers, 2017)Australia | Nursing homes | 20 family members | Family Interviews | Qualitative data | Family members of long-term care residents with dementia expressed positive perceptions of the Paro, perceiving that it improved mood, reduced agitation, and provided opportunity for communication for their relative. | The current cost of Paro was identified by family members as a major limitation to use |
|  | (Moyle, Bramble, Jones, & Murfield, 2018)  Australia | Nursing homes | 20 facility care staff | Staff interview | Qualitative data | Increasing excitement for Paro and decreasing enthusiasm for Plush Toy; value and function of Paro; opportunities for engagement; and alternatives vs. robustness. | Staff participants expressed concern that the cost of Paro could reduce opportunities for use within aged care |
|  | (Petersen, Houston, Qin, Tague, & Studley, 2017)  US | Dementia units | 61 participants (14 men) mean age  84.3 years. All participants were  diagnosed with dementia | Supervised group  interaction with Paro or  other activity (music,  physical activity and mental  stimulation). Randomized  controlled trial. Three sessions (20 min)/  week for 20 weeks | RAID, CSDD, GLDS,  pulse rate, pulse  oximetry, GSR and  medication | Anxiety scores,  depression scores and  pulse rate in Paro group all  significantly decreased over the study period compared  with control group. | Treatment with the PARO robot decreased stress and anxiety in the treatment group and resulted in reductions in the use of psychoactive medications and pain medications in elderly clients with dementia |
|  | (Robinson et al., 2013)  New Zealand | Retirement Home | 34 participants, aged >55 years | Group or individual  interaction with Paro  or alternative activity.  Randomised controlled trial. Two sessions (1 hour)/  week for 12 weeks | UCLA LS, GDS, QOLAD,  interview questionnaire  and observations | Loneliness scores  significantly decrease in the Paro group compared with control.  Residents enjoyed  sharing, interacting and  talking about Paro | Social robots in dementia care settings have to be simple and easy to use as well as stimulating and entertaining. |
|  | (Robinson, Macdonald, & Broadbent, 2015)  New Zealand | Residential care | 21 participants (seven men) mean  age 84.9 years | Supervised one-on-one  interaction with Paro. Pilot  study. One session (10 min) | Blood pressure reading:  before during and after  interaction | 1. Significant reductions in  systolic and diastolic blood  pressure.  2. Reduced systolic blood  pressure was sustained after  Paro was taken away.  3. Reduced diastolic blood  pressure was not sustained  after Paro was taken away.  4. Data suggest average  heart rate decreased. | Interacting with a pet-like robot can reduce blood pressure |
|  | (Roger, Guse, Mordoch, & Osterreicher, 2012)  Canada | Nursing home | 3 residents in the 1^st^ study; not mentioned how many staff completed survey; 4 residents in the 3^rd^ study | Paro was used as part of a summer training program for students; completed three Paro-related pilot studies | Face scale to measure residents’ depression, staff survey,  videotaped interactions | The integration of social commitment robots may be clinically valuable for older, agitated persons living with dementia in long-term care settings. | To provide training opportunities for long-term care staff so that they are better able to engage Paro in clinical interventions and increase a sense of personhood for persons living with dementia.  To include family perceptions and needs in the development of interventions using Paro, so that family involvement with persons living with dementia can be enhanced. |
|  | (Šabanovic, Bennett, Chang, & Huber, 2013)  US | Long-term care | Seven participants with dementia | Supervised group  interaction with Paro. Pilot  study. One session (30–45 min)/  week for 7 weeks | Observed behaviour  of primary and non-primary  interactor seen  in video-recording | 1. PARO increases activity  in particular modalities of  social interaction, which vary between primary and non-primary  interactors.  2. PARO improved activity  levels. | PARO’s positive effects on older adults’ activity levels show steady growth over the duration of study, suggesting Paro offered more than “novelty effect” |
|  | (Sung, Chang, Chin, & Lee, 2015)  Taiwan | Residential care | 12 participants (nine men) | Supervised group  interaction with Paro. Pilot study. Two sessions (30 min)/  week for 4 weeks | ACIS, Activity  Participation Scale | 1. Significant improvement  in communication and  interaction skills.  2. Significant improvement  in activity participation | Even short-term interactions signiﬁcantly improved the communication and interaction skills of older adults in residential care  Those with mild/moderate dementia who express their demand of communication more than those with severe dementia. |
|  | (Takayanagi, Kirita, & Shibata, 2014)  Japan | Nursing home | 30 participants (19 with mild/moderate  dementia  +11 with severe dementia), mean age 84.9 years (mild/moderate), 87.5 years (severe) | Supervised one-on-one interaction with Paro and Stuffed Lion. Pilot study. One session (~15 min)  for each intervention per  participants, separated by  3–6 months | Observed behaviour  seen in video-recording | In both groups:  1. Participants talked more  frequently to PARO.  2. Showed more positive  emotional expressions with  PARO.  In mild/moderate group only:  1. Showed more negative  emotional expressions with  Lion.  2. Frequencies of touching  and stroking and frequencies  of talking to staff member  were higher with Lion.  In severe group only:  1. Showed neutral  expression more frequently  with Lion. | Introducing PARO may increase willingness of the staff members to communicate and work with elderly people with dementia, especially those with mild/moderate dementia who express their demand of communication more than those with severe dementia. |
|  | (Thodberg et al., 2016)  Denmark | Nursing home | 100 participants with a mean age of  85.5 years | Supervised one-on-one  interaction with Paro, dog  or toy cat. RCT. Two sessions (10 min)/week for 6 weeks | MMSE, GBS, GDS,  CAM, sleep data and  BMI | 1. Greater interaction with  Paro and dog compared  with toy.  2. Cognitive and  independence scores  worsened over study period  in all groups (P<0.05).  3. Depression scores  improved over study in all  group (P<0.05). | The type of animal matters. “We found that the immediate responses to, and interaction with, the visiting animal depended on the type of animal that was brought along. The dog and the seal triggered the most interaction, in the form of physical contact, eye contact and verbal communication |
|  | (Valentí Soler et al., 2015)  Spain | Nursing home | Phase 1: 101 participants (13 men), mean age 84.7 years  Phase 2: 110 participants (11 men), mean age 84.7.  All participants were diagnosed with dementia. | Randomised  controlled trial. Phase 1:  Supervised group therapy (cognitive, musical and physical) with Paro or  a humanoid robot NAO (speaks).  Phase 2:  Supervised group therapy  (cognitive, musical and  physical) with Paro or Dog. Two sessions (30–40 min)/week for 3 months | GDS, MMSE, MMSE,  NPI, APADEM-NH and  the QUALID | Decreased apathy in both NAO and Paro groups  Increased delusions in the group treated with the humanoid robot  Increase QUALID scores  in the Paro group  Decreased night-time  behaviour disturbances in  the Paro group compared  with dog group | Robots have less needs for space, time, or care. Their sensors can respond to environmental changes (movements, sounds...) simulating interaction with the patient. They can monitor patients or be used in the therapy. Other potential beneﬁts of therapy with robots are that there are no known adverse eﬀects, specially trained personnel are not required and they can repeat the script in the same way as many times as it is required. |
|  | (Valentí Soler et al., 2015)  Spain | Day care | Phase 1: 20 participants (10 men),  mean age 77.9 years  Phase 2: 17 participants (eight men),  mean age 79 years  All participants were diagnosed with  dementia | Phase 1: Supervised group therapy  (cognitive and physical) with NAO.  Phase 2: Supervised group therapy  (cognitive and physical) with Paro. Crossover study. Two sessions (30–40 min)/week for 3 months | GDS, MMSE, MMSE,  NPI and QUALID | Patients with moderate/severe dementia cared for at a day care center, participants showed improvements in irritability and global neuropsychiatric symptoms after participating in sessions with the humanoid robot, but not after sessions with Paro. | The authors decided to focus on the use of humanoid robots in cognitive therapy for people with mild dementia and in the use of pet robots for people with moderate to severe dementia. |
|  | (Wada & Shibata, 2008)  Japan | Residential care | 12 participants, aged 67–89 years,  with mixed cognitive function | Free individual/  group interaction with Paro.  Pilot study. One session (9.5 hours)/  day for 4 weeks | Urinary tests, interviews  and video recording  observation | Increase in social  interaction and density of  social networks.  Improvement of participants’  vital organs reaction to  stress. | A lack of social and community ties results in deterioration of health. Paro broke through their mental barriers, made people with dementia talk again, with Paro and others |
|  | (Wada, Shibata, Saito, & Tanie, 2004)  Japan | Day care | 23 participants All of them were women, aged between 73 and 93 years old.  + 6 nursing staff | Paro was given to the elderly people at the day service center three days per week for ﬁve weeks. | face scale, POMS questionnaires,  urinary tests and  nursing comments, burnout scale | Improvement in mood  and reduction in depression  and dejection levels in both  groups.  Urinary results suggest  Paro interaction reduces  stress. | Staff burnout scores decreased after introducing Paro to the elderly people. Further work is needed to understand the relationship between patient mood and staff burnout |
|  | (Wada, Shibata, Saito, Sakamoto, & Tanie, 2005)  Japan | Long-term care | 14 participants (all female) aged 77–98 years | Free group interaction with Paro. Pilot study. Two sessions (1 hour)/  week for 1 year (and a  5-year follow-up) | Face scale, GDS and  nursing comments | A tendency to improve  depression after 8 weeks.  Improvement in mood.  Patients did not lose their interest in the long term | The elderly did not lose interest, and its effect on them showed up through one year  No breakdown and accident occurred by now. Paro fulfill its durability and safety of the robot |

ABMI, Agitated Behaviours Mapping Instrument; ACIS, Assessment of Communication and Interaction Skills; AES, Apathy Evaluation Scale; AI, Apathy Inventory; AIBO, Artificial Intelligence Robot; AOKLS, Ando Osada

and Kodama Loneliness Scale; APADEM-NH, Apathy Scale for Institutionalized Patients with Dementia Nursing Home version; APG, Accelerated Plethysmography; BARS, Brief Agitation Rating Scale; BMI, body mass

index; CAM, Confusion Assessment Method; CDR, Clinical Dementia Rating Scale; CgA, Chromogranin A; CMAI, Cohen Mansfield Agitation Inventory; Coop/Wonca, Mood scale; CSDD, Cornell Scale for Symptoms of

Depression in Dementia; GBS, Gottfries-Br.ne-Steen Scale; GDS, Geriatric Depression Scale; GLDS, Global Deterioration Scale; GSR, Galvanic Skin Response; IPPA, Goal attainment scale; LAPS, Lexington Attachment

to Pets Scale; LMBS, Lawton’s Modified Behaviour Stream; MMSE, Mini Mental State Examination; NPI, Neuropsychiatric Inventory; OERS, Observed Emotion Rating Scale; POMS, Profile of Mood States; QoLAD,

Quality of Life in Alzheimer’s Disease Scale; QUALID, Quality of Life Scale; RAID, Rating Anxiety in Dementia Scale; RUDAS, Rowland Universal Dementia Assessment Scale; SF-36, Short Form Health Survey; sMMSE, Severe Mini Mental State Examination; TAU, treatment as usual; UCLA LS, University of California Los Angeles Loneliness Scale.

References

Bemelmans, R., Gelderblom, G. J., Jonker, P., de Witte, L., Bemelmans Msc, R., Gelderblom Phd, G. J., … Phd, W. (2015). Effectiveness of Robot Paro in Intramural Psychogeriatric Care: A Multicenter Quasi-Experimental Study. *Journal of the American Medical Directors Association*, *16*, 946–950. http://doi.org/10.1016/j.jamda.2015.05.007

Bemelmans, R., Gelderblom, G. J., Jonker, P., de Witte, L., Jan Gelderblom, G., Jonker, P., & de Witte, L. (2016). How to use robot interventions in intramural psychogeriatric care; A feasibility study. *Applied Nursing Research*, *30*, 154–157. http://doi.org/10.1016/j.apnr.2015.07.003

Dodds, P., Martyn, K., & Brown, M. (2018). Infection prevention and control challenges of using a therapeutic robot. *Nursing Older People*. http://doi.org/http://dx.doi.org/10.7748/nop.2018.e994

Iacono, I., & Marti, P. (2016). Narratives and emotions in seniors affected by dementia: A comparative study using a robot and a toy. In *25th IEEE International Symposium on Robot and Human Interactive Communication, RO-MAN 2016*. http://doi.org/10.1109/ROMAN.2016.7745149

Jones, C., Moyle, W., Murfield, J., Draper, B., Shum, D., Beattie, E., & Thalib, L. (2018). Does Cognitive Impairment and Agitation in Dementia Influence Intervention Effectiveness? Findings From a Cluster-RCT With the Therapeutic Robot, PARO. *Journal of the American Medical Directors Association*. http://doi.org/10.1016/j.jamda.2018.02.014

Jøranson, N., Pedersen, I., Rokstad, A. M. M., Aamodt, G., Olsen, C., Ihlebæk, C., … Ihlebaek, C. (2016). Group activity with Paro in nursing homes: systematic investigation of behaviors in participants. *International Psychogeriatrics C International Psychogeriatric Association*, *28*(8), 1345–1354. http://doi.org/10.1017/S1041610216000120

Jøranson, N., Pedersen, I., Rokstad, A. M. M., & Ihlebæk, C. (2015). Effects on Symptoms of Agitation and Depression in Persons With Dementia Participating in Robot-Assisted Activity: A Cluster-Randomized Controlled Trial. *Journal of the American Medical Directors Association*. http://doi.org/10.1016/j.jamda.2015.05.002

Joranson, N., Pedersen, I., Rokstad, A. M. M., Ihlebaek, C., Jøranson, N., Pedersen, I., … Ihlebæk, C. (2016). Change in quality of life in older people with dementia participating in Paro-activity: a cluster-randomized controlled trial. *Journal of Advanced Nursing*, *72*(12), 3020–3033. http://doi.org/10.1111/jan.13076

Kidd, C. D., Taggart, W., & Turkle, S. (2006). A sociable robot to encourage social interaction among the elderly. *Proceedings - IEEE International Conference on Robotics and Automation*, *2006*, 3972–3976. http://doi.org/10.1109/ROBOT.2006.1642311

Lane, G. W., Noronha, D., Rivera, A., Craig, K., Yee, C., Mills, B., & Villanueva, E. (2016). Effectiveness of a social robot, “Paro,” in a VA long-term care setting. *Psychological Services*. http://doi.org/10.1037/ser0000080

Marti, P., Bacigalupo, M., Giusti, L., Mennecozzi, C., & Shibata, T. (2006). Socially Assistive Robotics in the treatment of behavioural and psychological symptoms of dementia. *Proceedings of the First IEEE/RAS-EMBS International Conference on Biomedical Robotics and Biomechatronics, 2006, BioRob 2006*, *2006*(October), 483–488. http://doi.org/10.1109/BIOROB.2006.1639135

Mervin, M. C., Moyle, W., Jones, C., Murfield, J., Draper, B., Beattie, E., … Thalib, L. (2018). The Cost-Effectiveness of Using PARO, a Therapeutic Robotic Seal, to Reduce Agitation and Medication Use in Dementia: Findings from a Cluster-Randomized Controlled Trial. *Journal of the American Medical Directors Association*, 1–5. http://doi.org/10.1016/j.jamda.2017.10.008

Moyle, W., Bramble, M., Jones, C. J., Murfield, J. E., & Bowers, B. J. (2017). “She Had a Smile on Her Face as Wide as the Great Australian Bite”: A Qualitative Examination of Family Perceptions of a Therapeutic Robot and a Plush Toy. *Gerontologist*, *00*(00), 1–9. http://doi.org/10.1093/geront/gnx180

Moyle, W., Bramble, M., Jones, C., & Murfield, J. (2018). Care staff perceptions of a social robot called Paro and a look-alike Plush Toy: a descriptive qualitative approach. *Aging and Mental Health*. http://doi.org/10.1080/13607863.2016.1262820

Moyle, W., Cooke, M., Beattie, E., Jones, C., Klein, B., Cook, G., & Gray, C. (2013). Exploring the Effect of Companion Robots on Emotional Expression in Older Adults with Dementia: A Pilot Randomized Controlled Trial. *Journal of Gerontological Nursing*, *39*(5), 46–53. Retrieved from https://www-healio-com.ezproxy.library.ubc.ca/nursing/journals/jgn/2013-5-39-5/%7B4148aa00-206b-4ba9-9e37-d730b6aecb90%7D/exploring-the-effect-of-companion-robots-on-emotional-expression-in-older-adults-with-dementia-a-pilot-randomized-controlled-trial.pd

Moyle, W., Jones, C. J., Murfield, J. E., Thalib, L., Beattie, E. R. R. A., Shum, D. K. K. H., … Draper, B. M. (2017). Use of a Robotic Seal as a Therapeutic Tool to Improve Dementia Symptoms: A Cluster-Randomized Controlled Trial. *Journal of the American Medical Directors Association*, *18*, 766–773. http://doi.org/10.1016/j.jamda.2017.03.018

Petersen, S., Houston, S., Qin, H., Tague, C., & Studley, J. (2017). The Utilization of Robotic Pets in Dementia Care. *Journal of Alzheimer’s Disease*, *55*(2), 569–574. http://doi.org/10.3233/JAD-160703

Robinson, H., MacDonald, B. A., Kerse, N., Broadbent, E., Pgdipsci, H. R., Macdonald Phd, B. A., … Broadbent, E. (2013). Suitability of Healthcare Robots for a Dementia Unit and Suggested Improvements. *Journal of the American Medical Directors Association*, *14*, 34–40. http://doi.org/10.1016/j.jamda.2012.09.006

Robinson, H., Macdonald, B., & Broadbent, E. (2015). Physiological effects of a companion robot on blood pressure of older people in residential care facility: A pilot study. *Australasian Journal on Ageing*, *34*(1), 27–32. http://doi.org/10.1111/ajag.12099

Roger, K., Guse, L., Mordoch, E., & Osterreicher, A. (2012). Social commitment robots and dementia. *Canadian Journal on Aging*, *31*(1), 87–94. http://doi.org/10.1017/S0714980811000663

Šabanovic, S., Bennett, C. C., Chang, W. L., & Huber, L. (2013). PARO robot affects diverse interaction modalities in group sensory therapy for older adults with dementia. In *IEEE International Conference on Rehabilitation Robotics*. http://doi.org/10.1109/ICORR.2013.6650427

Sung, H.-C., Chang, S.-M., Chin, M.-Y., & Lee, W.-L. (2015). Robot-assisted therapy for improving social interactions and activity participation among institutionalized older adults: A pilot study. *Asia-Pacific Psychiatry*, *7*(1), 1–6. http://doi.org/10.1111/appy.12131

Takayanagi, K., Kirita, T., & Shibata, T. (2014). Comparison of verbal and emotional responses of elderly people with mild/moderate dementia and those with severe dementia in responses to seal robot, PARO. *Frontiers in Aging Neuroscience*. http://doi.org/10.3389/fnagi.2014.00257

Thodberg, K., Sørensen, L. U., Videbech, P. B., Poulsen, P. H., Houbak, B., Damgaard, V., … Christensen, J. W. (2016). Behavioral responses of nursing home residents to visits from a person with a dog, a robot seal or atoy cat. *Anthrozoos*, *29*(1), 107–121. http://doi.org/10.1080/08927936.2015.1089011

Valentí Soler, M., Agüera-Ortiz, L., Olazarán Rodríguez, J., Mendoza Rebolledo, C., Pérez Muñoz, A., Rodríguez Pérez, I., … Martinez Martin, P. (2015). Social robots in advanced dementia. *Frontiers in Aging Neuroscience*, *7*(JUN). http://doi.org/10.3389/fnagi.2015.00133

Wada, K., & Shibata, T. (2008). Social and physiological influences of robot therapy in a care house. *Interaction Studies*, *9*(2), 258–276. http://doi.org/10.1075/is.9.2.06wad

Wada, K., Shibata, T., Saito, T., Sakamoto, K., & Tanie, K. (2005). Psychological and Social Effects of One Year Robot Assisted Activity on Elderly People at a Health Service Facility for the Aged. *Proceedings of the IEEE International Conference on Robotics and Automation (ICRA)*, (April), 2785–2790. http://doi.org/10.1109/ROBOT.2005.1570535

Wada, K., Shibata, T., Saito, T., & Tanie, K. (2004). Effects of robot-assisted activity for elderly people and nurses at a day service center. *Proceedings of the IEEE*, *92*(11), 1780–1788. http://doi.org/10.1109/JPROC.2004.835378
